# A pilot study of transrectal endoscopic ultrasound elastography in inflammatory bowel disease

**DOI:** 10.1186/1471-230X-11-113

**Published:** 2011-10-20

**Authors:** Nadan Rustemovic, Silvija Cukovic-Cavka, Marko Brinar, Davor Radić, Milorad Opacic, Rajko Ostojic, Boris Vucelic

**Affiliations:** 1 Department of Gastroenterology University Hospital Centre Zagreb, Croatia

**Keywords:** Crohn's disease, ulcerative colitis, elastography, ultrasound

## Abstract

**Background:**

Using standard diagnostic algorithms it is not always possible to establish the correct phenotype of inflammatory bowel disease which is essential for therapeutical decisions. Endoscopic ultrasound elastography is a new endoscopic procedure which can differentiate the stiffness of normal and pathological tissue by ultrasound. Therefore, we aimed to investigate the role of transrectal ultrasound elastography in distiction between Crohn's disease and ulcerative colitis.

**Methods:**

A total 30 Crohn's disease, 25 ulcerative colitis, and 28 non-inflammatory bowel disease controls were included. Transrectal ultrasound elastography was performed in all patients and controls. In all ulcerative coltis patients and 80% of Crohn's disease patients endoscopy was performed to assess disease activity in the rectum.

**Results:**

Significant difference in rectal wall thickness and strain ratio was detected between patients with Crohn's disease and controls (p = 0.0001). CD patients with active disease had higher strain ratio than patients in remission (p = 0.02). In ulcerative colitis group a significant difference in rectal wall thickness was found between controls and patients with active disease (p = 0.03). A significant difference in rectal wall thickness (p = 0.02) and strain ratio (p = 0.0001) was detected between Crohn's disease and ulcerative colitis patient group. Crohn's disease patients with active disease had a significantly higher strain ratio compared to ulcerative colitis patients with active disease (p = 0.0001).

**Conclusion:**

Transrectal ultrasound elastography seems to be a promising new diagnostic tool in the field of inflammatory bowel disease. Further study on a larger cohort of patients is needed to definitely assess the role of transrectal ultrasound elastography in inflammatory bowel disease.

## Background

The diagnosis of inflammatory bowel disease (IBD) is based on clinical, endoscopic, radiologic and histologic criteria [1]. There are two main IBD phenotypes - Crohn's disease (CD) and ulcerative colitis (UC). In some circumstances, especially when disease extension is restricted exclusively to colon or in acute, severe phases of the disease like pancolitis when inflammation is widely spread to the colon, establishing an accurate diagnosis is very difficult. Assigning the exact phenotype of IBD as Crohn's colitis or UC is essential for guiding therapy decisions and detecting complications that warrant treatment and when IBD is confined to the colon it is especially important to define final phenotype before surgical decision.

Endoscopic examination is the mainstay in the diagnosis of IBD. Endoscopic appearance (distribution and shape of lesions) helps to differentiate CD from UC in most cases. Histology analysis confirms the elements of chronic inflammation but it is not exclusively diagnostic. Patients with UC may have atypical histological features such as microscopic inflammation of the ileum, patchiness, and relative rectal sparing at the time of diagnosis. These findings should not prompt the clinician to change the diagnosis from UC to CD. Other endoscopic findings, such as macroscopic cobblestoning, segmental colitis, ileal stenosis and ulceration, perianal disease, and pathologically confirmed multiple granulomas in the small bowel or colon more strongly suggest a diagnosis of CD.

Unfortunately, none of the classic or new endoscopic tools is strictly diagnostic for the exact IBD phenotype. The clinicians hope that progress being made in genetics, serological markers, and imaging studies will lead to more reliable determination of exact IBD phenotype [2]. In the meantime it is reasonable to examine all possibilities to find better methods for differentiation between distinct IBD phenotypes. We think that endoscopic ultrasound (EUS) elastography is a perspective and promising method to achieve this goal. It is a new endoscopic procedure which can differentiate the stiffness of normal and pathological tissue by ultrasound. This finding is based on B-mode scanning during compressions [3]. There are some data on elastography applied on the GI tract, biliary tract, kidney, muscle, breast and the heart [3-5]. According to the fact that imaging of ultrasound tissue elasticity is a way to distinct characteristics of tissue we hypothesized that EUS elastography has the role in assessing the thickness of bowel wal in patients with IBD. As elastography has a potential to define tissue characteristics we hypothesized that using this method we can differentiate two forms of inflammation in colon: Crohn colitis and ulcerative colitis [6,7].

Based on the fact that CD is a transmural disease while in UC inflammation is limited to the mucosa and submucoasa we aimed to assess the role of transrectal ultrasound elastography in distinction between CD and UC.

## Methods

We included a total of 55 patients, 30 with CD and 25 with UC, and 28 non-IBD controls. Diagnosis of CD and UC was confirmed by clinical, radiologic, endoscopic, and histologic criteria. All biopsies of patients were reviewed by our pathologist who is well-experienced in IBD. Patients in whom there was doubt about the disease phenotype (unclassified IBD (IBDU))) were excluded from this study.

**Figure 1:**
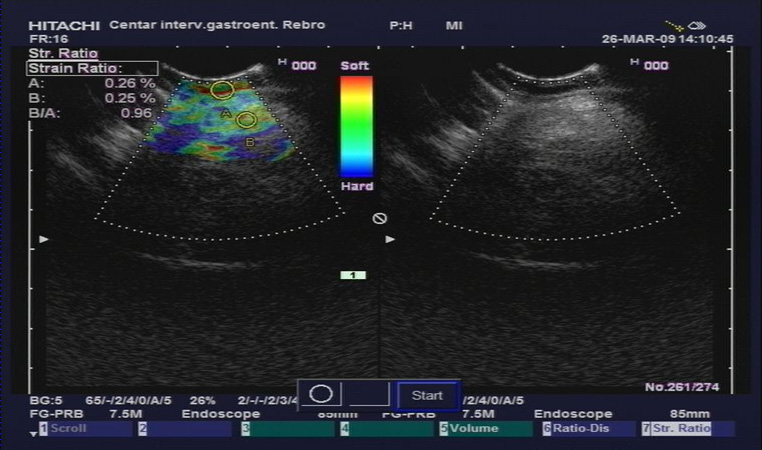
**TRUS elastography in a UC patient**. Letter "A" represents first region of interest in mucosal tissue. Letter "B" represents second region of interest in perirectal tissue.

**Figure 2:**
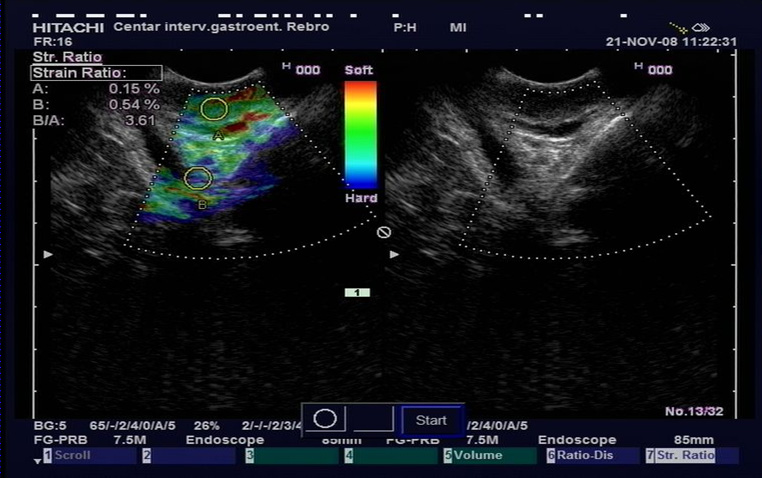
**TRUS elastography in a CD patient**. Letter "A" represents first region of interest in mucosal tissue. Letter "B" represents second region of interest in perirectal tissue.

Clinical characteristics of CD and UC patients are given in table 1. As can be seen 46% of included CD patients had rectal involvement either at endoscopy or described in previous medical history. In all UC patients and in 80% of CD patients (24/30) endoscopy was performed to assess disease activity in the rectum. For UC, disease activity was assessed based on the Baron score [8]. For CD patients disease activity in the rectum was graded by the endoscopist based on the endoscopic appearance as "remission" if no visible lesions were seen, as "mild activity" if erythematous mucosa and/or erosions of the mucosa were seen but with no ulcers in the rectum and as "severe activity" if ulcerations or spontaneous bleeding were found. In IBD patients endoscopy was performed after a median of 0.16 months, interquartile range (IQR) [0.03-0.99] months, from TRUS. In all patients and non-IBD controls transrectal ultrasound (TRUS) elastography was performed. Linear echo-endoscope (Pentax FG-38 UX) with the probes of 7, 5-12 MHz (Hitachi EUB 8500) was used for the investigation. All patients were examined in the left lateral decubital position, without previous preparation. The probe was covered with condom and inserted in the ampulla of the rectum under direct vision. A 170 degree linear probe display of the rectal wall and surrounding tissue was provided. Total wall thickness was measured. Real time tissue elastography was performed. Quantification of elastography data was evaluated by the strain ratio (SR). In order to obtain EUS elastography strain ratio an elipse was adjusted to the rectal wall, and second one (same diameter) was adjusted to the surrounding tissue up to 15 mm from the rectal wall where the elastography signal was most obvious. Strain ratio is ratio of strain between two regions of interest (ROI) in the same image. Rectal wall tissue was used as a first ROI, and perirectal tissue as second (Figure 1 and 2). The SR (rectal wall tissue strain %/perirectal tissue strain %) was calculated automaticaly using Hitachi EUB-8500 software.

**Table 1:** Clinical characteristics of CD and UC patients

	CD	UC
Number of patients	30	25
Sex - no. of patients (%)		
Female	19 (63, 3)	15 (60, 0)
Male	11 (36, 7)	10 (40, 0)
Age at diagnosis (years), median [interquartile range]	23, 01 [18,01-33,27]	33, 35 [23,08-46, 03]
Age at TRUS (years), median [interquartile range]	30, 64 [23,67-42,37]	38, 37 [26,15-53,07]
Disease duration to TRUS (years), median [interquartile range]	5,15[2,02-13,01]	5, 83 [1,82-9,38]
Localization - no. of patients (%)		
L1 ± UGI	9 (30)	
L2 ± UGI	4 (13, 3)	
L3 ± UGI	17 (56, 7)	
Rectum involved	14 (46, 7)	
Proctitis		3 (12, 0)
Left sided colitis		9 (36, 0)
Pancolitis		13 (52, 0)
Behaviour* - no. of patients (%)		
B1 ± perianal	14 (50)	
Stricturing ± perianal	9 (32, 1)	
Penetrating ± perianal	5 (17, 9)	
Perianal (any)	12 (40)	
Endoscopy at time of TRUS - no. of patients (%)	24 (80)	25 (100)
Inactive	10 (43, 5)	
Mild activity	9 (34, 8)	
Severe activity	5 (21, 7)	
Baron score 0		11 (11, 8)
Baron score 1		3 (17, 6)
Baron score 2		11 (70, 6)
Baron score 3		0 (0)

* Information about biheviour was available in 28 patients

SR was measured 3 times in anterior, posterior and lateral region ofthe rectal wall and surrounding tissues. Middle value was used in statistical analysis [9]. All examinations were performed by one observer.

Informed consent was obtained from all patients and the study was approved by the ethics committee of the University Hospital Centre Zagreb.

### Statistics

Basic group comparisons were performed using SPSS ver. 15.0 for Windows (SPSS Inc, Chicago, IL). Differences in continuous variables between IBD patients and non-IBD controls as well as between CD and UC patients were tested using independent sample t test and Mann Whitney U test for normally and not normally distributed variables respectively. Significance was accepted at a level of p < 0.05.

## Results

**Figure 3:**
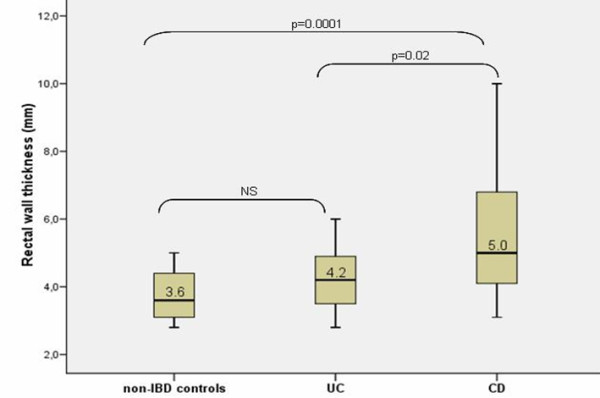
**Rectal wal thickness in non-IBD controls, CD and UC patients**. Box plots indicate 25th and 75th percentile. Medians are represented by straight lines. Error bars indicate 5th and 95th percentiles.

**Figure 4:**
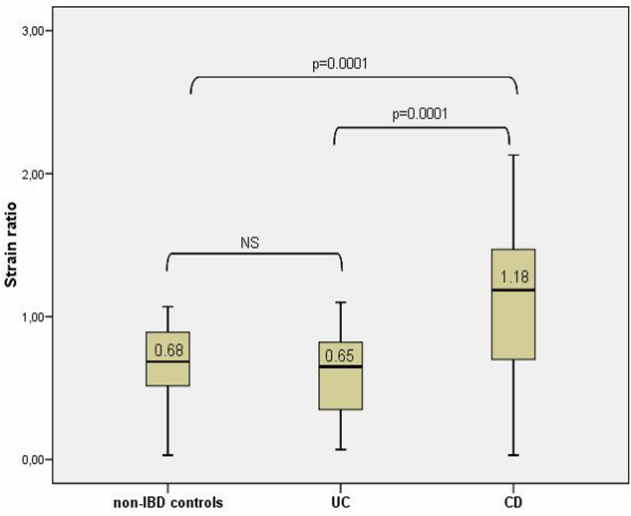
**Strain ratio of non-IBD controls, CD and UC patients**. Box plots indicate 25th and 75th percentile. Medians are represented by straight lines. Error bars indicate 5th and 95th percentiles.

A summary of results is given in table 2 and Figures 3 and 4. As can be seen in table 2 a significant difference in rectal wall thickness between IBD group as a whole and non IBD controls was found (p = 0.001). We also detected a trend toward a higher strain ratio in IBD patients as compared to controls but the difference did not reach significance. IBD patients were significantly younger than non-IBD controls when they underwent TRUS (p = 0.0001). As can be seen in Figure 3 and 4, a significant difference was found both rectal wall thickness and strain ratio in CD patient group compared to controls (p = 0.0001). Interestingly, even the CD patients without rectal involvement had a significantly thicker rectal wall when compared to controls (median 4.65 mm vs 3.6 mm; p = 0.028). In the CD patient group patients with active disease (n = 14) had a significantly higher strain ratio in comparison with patients in remission (n = 10) (p = 0.02; median 1.37 IQR [1.20-1.56] vs median 0.97 IQR [0.54-1.20]). No significant difference in rectal wall thickness was found in patients with active CD and CD patients in remission. In the UC group no significant difference was found between UC patients and non-IBD controls in regards to rectal wall thickness and strain ratio (Figure 3 and 4). The subgroup of UC patients with active inflammation in the rectum on endoscopy had a significantly thicker rectal wall when compared to controls (median 4.5 mm vs 3.6 mm; p = 0.03). No significant difference in strain ratio was detected. In the UC patient group no significant difference in rectal wall thickness and strain ratio was found between UC patients with active disease (n = 14) and UC patients in remission (n = 11). Comparing the group of CD and UC patients we found a significant difference in age at diagnosis, rectal wall thickness and strain ratio between the groups with CD patients being significantly younger at diagnosis (median 23.01 years vs 33.35 years; p = 0.016) and having significantly thicker rectal wall (median 5.0 mm vs 4.2 mm; p = 0.021) and higher strain ratio (1.18 vs 0.65; p = 0.0001) in comparison with the UC patient group. No significant difference in age at TRUS and disease duration to TRUS was detected between the groups. Comparing active CD and UC patients we detected a significantly higher strain ratio in the group of active CD patients when compared to active UC patients (median 1.30 vs 0.49; p = 0.0001). No significant difference in rectal wall thickness was detected.

**Table 2:** Summary of results.

	IBD	Non-IBD controls	P value
Age at TRUS, median [IQR], years	33.97 [25.04-46.86]	59.11 [46.51-73.03]	0.0001

Rectal wall thickness, median [IQR]	4, 85 [3.7-5.9]	3.6 [3.1-4.4]	0.001

Strain ratio, median [IQR]	0.82 [0.5-1.23]	0.68 [0.51-0.89]	0.06

## Discussion

There are numeorus papers in the current literature on the issue of EUS elastography. The method was initialy inaugurated to distinguish te benign from malignant pancreatic lesions [6,10,11]. Although it can't replace biopsy and histological confirmation of cancer, „virtual biopsy" done by EUS elastography could provide good information of the consistency of the tissue of interest [12].

In this study we hypothesized that „virtual biopsy" technique might have a good implementation in differentiating colonic tissue in CD and UC [4,13].

Namely, phenotyping of the IBD in clinical practice can be a very difficult problem. Sometimes the pathological diagnosis is insufficient or uncertain but also one form of the disease can change over the years. An adequate phenotyping could have a major impact on therapy and prognosis for the patients. Differentiating UC from Crohn's colitis may contribute to inaccurate diagnosis in up to 30% of IBD patients. The main reason for this diagnostic uncertainty is overlapping of clinical and histological features [14]. This fact has a great impact in everyday practice because prolongation in definite diagnosis can lead to delay in initiation of appropriate treatment. Different phenotypes require specific diagnostic and therapeutic solutions [15]. Phenotyping is very important because of three specific reasons. First, Crohn's colitis and UC have different risk of complications (fistulas, strictures, extraintestinal manifestations) that require a different therapeutic approach. Second, regarding to drugs, there is no evidence that mesalazine is effective in active Crohn's colitis while quite the contrary ulcerative colitis should be treated first with mesalazine [16,17]. Delay of introduction of immunosupressives in misdiagnosed patients with Crohn's colitis can be deleterious. Third, accurate assessment of disease is important in the case of the need for a definite decision of surgical intervention such as proctocolectomy. Specifically, the formation of ileoanal pelvic reservoir in case of unrecognized CD is more often complicated by fistula, stenosis and pelvic sepsis [18].

Biomarkers are increasingly being proposed to improve phenotyping of patients [19-21]. Unfortunatelly, in spite of many different new studies on serologic and genetic markers, there is no satisfactory panel of tests which can ultimately confirm the diagnosis. Therefore it's necessary to search for new diagnostic procedures that could contribute to more accurate recognition of the disease phenotype and improve diagnostic confidence in IBD. In this sense, EUS elastography could be an option as a new diagnostic procedure which has the potential to recognize differences in the UC tissue and Crohn's colitis tissue.

In order to define clear differences between the wall of the colon in CD and UC, our investigation was focused on the characteristics of the rectal wall and perirectal tissue, observed by the EUS elastography with SR calculation. Perirectal tissue in UC patients is supposed to be soft, without inflammation, and in CD patients „hard tissue" reflects the transmural nature of inflammation. These qualitative and quantitative elastography data could lead to differentiation of IBD phenotypes, and could help in clinical decision making in patients with IBD unclassified (IBDU).

In our study we detected a significant difference in rectal wall thickness when comparing IBD group as a whole and controls which is in agreement with other reports from the literature [22,23]. Interestingly, we also found a significant difference in rectal wall thickness between CD patients without rectal involvement and controls. The significance of this finding in our pilot study is unclear but it could suggest a possible predictive role of TRUS elastography in CD. Bearing in mind the fact that CD can involve any part of the GI tract it would be interesting to identify such patients early in the course of the disease and follow them prospectively to see whether rectal involvement or perianal disease will develop. In UC patients a significant difference in rectal wall thickness but not strain ratio was found between active UC patients and controls. This finding reflects the fact that inflammatory process in UC is confined to the mucosa and submucosa leading to the thickening of the rectal wall in acute inflammation but without changes in perirectal tissue as measured by strain ratio. A significant difference was detected in rectal wall thickness and strain ratio between CD and UC patient group reflecting the difference in pathogenetic mechanisms driving these diseases with CD being characterized by transmural inflammation as opposed to UC. Finally, we detected a significant difference in both rectal wall thickness and strain ratio between CD patients with rectal involvement and UC patients with active disease.

According to age, we detected a significant difference in age at TRUS between IBD group and control group. However, we believe that this difference did not have any influence on our results as, to the authors' knowledge, there are no data in the literature showing that rectal wall thickness changes with age [24,25]. However, we believe that the results of our study can be useful in determining the exact phenotype of IBD. Accurate phenotyping is of crucial importance in guiding therapeutic decisions, especially for planning the type of surgical procedure. However, currently about 10% of patients with inflammation restricted to the colon can not be accurately assessed using standard diagnostic techniques [26,27]. More important, in 4-6% of UC patients undergoing proctocolectomy with ileoanal pouch formation CD is subsequently diagnosed with significant morbidity and high rate of pouch failure [28,29]. Our findings suggest that TRUS elastography might play a role in determining the phenotype in IBD patients who can not be classified using standard diagnostic tools. Although we found a significant difference in rectal wall thickness and significant difference in strain ratio between CD patients with rectal involvement and active UC patients our study is limited with a small number of patients included. A prospective study with inclusion of a greater number of patients and ultimately construction of receiver operating characteristics (ROC) curve is needed to definitely assess the value of TRUS elastography in IBD.

## Conclusion

TRUS elastography with strain rato calculation provides valuable information regarding the stiffness of the rectal and perirectal tissue and can help to differentiate CD from UC. According to our results we can conclude that TRUS elastography could be one of the perspective and promising diagnostic tools in IBD. A prospective study on a large cohort of patient is necessary to consolidate and confirm the results and establish the role of TRUS in distinguishing Crohn's colitis and UC. In addition, one of the important benefits of EUS elastography in the long run could be the possibility of identifying individuals at risk of developing a transmural disease, thereby facilitating appropriate action for prevention of disease complications.

## Competing interests

The authors declare that they have no competing interests.

## Authors' contributions

NR conceived and designed the study, performed TRUS elastography and drafted the manuscript. SCC participated in the design of the study and helped in drafting the manuscript. MB collected the clinical data, performed statistical analysis and helped in drafting the manuscript. DR participated in the design of the study and collected clinical data. MO participated in the design of the study and critically revised the manuscript. RO participated in the design of the study and critically revised the manuscript. BV participated in the design of the study, critically revised the manuscript and approved the final version to be published. All authors read and approved the final manuscript.

## Pre-publication history

The pre-publication history for this paper can be accessed here:

http://www.biomedcentral.com/1471-230X/11/113/prepub
